# The J-shape of β2GPI reveals a cryptic discontinuous epitope across domains I and II

**DOI:** 10.1016/j.yjsbx.2025.100135

**Published:** 2025-08-20

**Authors:** C.J. Lalaurie, M. Kulke, N. Geist, M. Delcea, P.A. Dalby, T.C.R. McDonnell

**Affiliations:** a Division of Medicine, Rayne Building, 5 University Street, University College London WC1E 6JF London, United Kingdom; b Theoretical Biophysics, Ernst-Otto-Fischer-Str. 8, 85748 Garching b., Technical University Munich, Munich, Germany; c Biophysical Chemistry, Felix Hausdorff-Strasse 4, 17487, University of Greifswald, Greifswald, Germany; d Department of Biochemical Engineering, Bernard Katz, Gower Street, University College London WC1E 6BT London, United Kingdom

**Keywords:** Structural biology, Molecular Dynamics, Auto-antigen epitope, APS, Beta-2-glycoprotein I

## Abstract

Beta-2-Glycoprotein I is the main target for pathogenic antiphospholipid syndrome autoantibodies. It can adopt several conformations, including an O-shape and two more linear J- and S-shapes. The *in vivo* existence of the O-shape is debated, and doubt remains pertaining to the pathogenic impact of each shape. Studies have shown that APS antibodies react weakly with the O-shape and bind to the linear shapes due to the exposure of a cryptic epitope in the 1st domain. How the protein transitions from O-shape to the linear shapes remains unknown. While the main epitope is widely recognised as the R39-R43 peptide, there is evidence pointing to a discontinuous epitope across domains I and II (DI & DII). We used molecular dynamics simulations to examine the potential pathways of conformational shift from the O-shape to the open forms, and the impact of plasmin clipping on these pathways. Through these studies, starting in a theorised O-shape, we identified that peptides R39-R43, T50-N56 and R63-F67 become more exposed and have increased stability in the J- and S-shapes relative to the O-shape. These changes are likely due to a shift in DII of the T106-G109 loop, which twists to form contacts with the DI K33-Y36 loop. The R39-R43 peptide is brought closer to R63-F67 suggesting a more complex DI epitope than previously theorised. These effects were observed in the wild type and plasmin clipped model, with the effect being larger in the latter. These results are in good agreement with the increased antibody binding observed experimentally for the clipped protein. We therefore suggest that we have been able to identify the structural mechanism at the residue level which results in increased antibody binding in the J-Shape, and specifically in the clipped protein.

## Highlights

• Beta-2-glycoprotein I is the main target of auto-antibodies in anti-phospholipid syndrome (APS), however the complete epitope is not known.
• We present a thorough molecular dynamics study which has identified surface changes in key areas as the protein moves through these conformations.
• The results of our simulations explain the structural mechanism which reveals the cryptic epitope, hidden in the O-Shape, at the residue level.
• The results demonstrate the larger impact in the plasmin clipped protein, consistent with increased antibody response, as seen experimentally.
• By uncovering this mechanism, we can think about treatments targeting, or mimicking, these areas to protect the patients from their immune system.

## Graphical abstract

**Figure:**
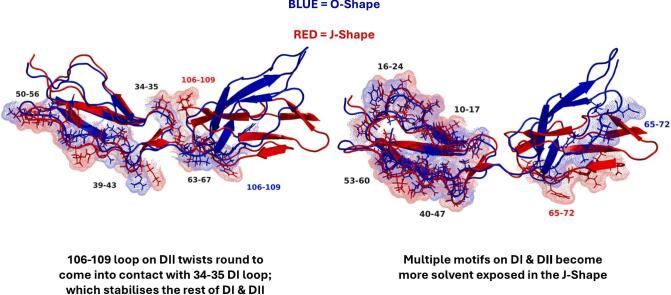

## Introduction

Beta-2-Glycoprotein I (β2GPI) is a soluble blood protein of ∼ 48 kDa comprised of five sequential domains, with a blood circulation level of ∼ 0.2 mg/mL (Aǧar, 2010). Domains I to IV (DI-DIV) are complement control protein (CCP)-like domains, while DV is a slightly larger, lysine-rich domain (Schwarzenbacher, 1999). β2GPI has a number of important functions within the body, including complement regulation and receptor recognition (Gropp, 2011, McDonnell, 2020, Agostinis, 2011), and has also been linked to several diseases, most prominently antiphospholipid syndrome (APS) (Zhang, 2016, Qi, 2016). There are currently three reported conformations of β2GPI: a J- or fishhook-shape (Ruben, 2020), an S-shape (Hammel, 2002) and a circular O-shape (Aǧar, 2010), identified by electron microscopy or small-angle X-ray scattering experiments. In the O-shape, DI and DV are close together and form an interface. It has been hypothesised that β2GPI is produced and circulates in the O-shape and extends to the J- and S-shapes upon binding to anionic surfaces such as phospholipids (Subang, 2000, Hamdan et al., 2007), however recent research has refuted this theory (Kumar et al., 2023). It should be noted that the purification method used in this study may be the cause for their observation, as pointed out by the authors in the article. It is also possible that despite being produced in the O-shape, this conformation is inherently unstable and spontaneously opens when released into circulation (Kumar et al., 2023).

The theory of binding and opening is supported by the observation that APS patients, for which the primary antigen site is located on DI (Iverson et al., 1998, Iverson, 2002, Reddel et al., 2000), do not present β2GPI-antibody complexes in circulating plasma (Aǧar, 2010) suggesting the epitope is not accessible in circulating forms. Moreover, it has previously been shown that the J-shape is significantly more prone to antibody binding relative to the O-shape (De Groot and Meijers, 2011, De Laat et al., 2006, De Laat, 2011). Differences in binding to hydrophobic and hydrophilic plates also suggested conformational changes in structure is required to promote antibody binding (De Laat et al., 2005). This suggests that the epitope, thought to be primarily within residues R39-R43 of DI, is made more solvent accessible in the J-shape allowing patient antibody binding. There is, however, debate about the exact nature of the epitope. Whilst binding to R39-R43 has been established in several assays (Iverson et al., 1998, Reddel et al., 2000, De Laat, 2011, Moghbel, 2022, Ioannou, 2007) there is some debate to the exact process of binding. It has for example been shown that APS patients’ antibodies interacted to a lesser extent with β2GPI mutated at the single K19, T50 and N56 sites within DI (Iverson, 2002). It has also been suggested that anti-β2GPI antibodies react to a motif rather than specific residues (de Moerloose, 2017), and that the epitope may be discontinuous and involve the DI-DII linker region (Ioannou, 2007). These residues may therefore be either directly involved in the antibody binding or affect the binding through cross-correlation effects to the epitope residues.

β2GPI is also a target for plasmin, which cleaves it within DV at K317-T318, removing eight C-terminal residues to leave a slightly shorter protein (Ohkura et al., 1998). This clipped form of β2GPI has been found at higher levels in the serum of APS patients compared to healthy controls (Matsuura, 2000, Kato, 2000). The exact effect of this on the behaviour and structure of β2GPI is not well characterised, though biophysical analyses have shown that clipped β2GPI leads to increased APS antibody binding and novel conformations (Matsuura, 2000). The clipped protein also has altered functionality compared to the wild type (WT) form (Yasuda, 2004, Sakai et al., 2007). The clipped form of β2GPI shows reduced binding to phospholipids at high NaCl concentrations, however in conditions matching human plasma tonicity (∼0.15 M) the binding affinity was found to remain very close to that of WT β2GPI (Ohkura et al., 1998).

**Fig. 1:**
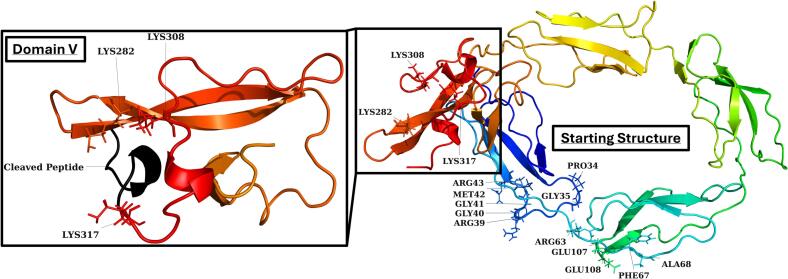
“Chainbow” representation of the starting structure. Zoomed visual of DV to highlight the cleaved peptide (318–326). For increased clarity the glycans used in the simulations are not shown here (see Supplementary Fig. S1).

Whilst there is some controversy about the dominant physical structure of β2GPI, the process of transitioning between these structures has remained completely unexplored. This may largely be due to the complexities in studying such a fast process, and due to the difficulties in modifying the structures themselves in ways which mirror the serum of patients and controls alike. By exploring how this transition occurs, and uncovering short-lived transition states in between the O-shape and J-shape, it may be possible to target new areas in order to prevent the full transition from occurring and reduce the overall pathogenicity of the protein *in vivo*. As such molecular simulation offers the potential to explore the process by which β2GPI opens from a circular shape through to its established linear forms. In this work, we utilised a theoretical model for the circular shape of β2GPI as the starting point for molecular dynamics simulations (MDS). A chainbow coloured cartoon representation of the starting structure (Fig. 1) highlights the key difference in DV, while Supplementary Fig. S1 shows the full structure including the four glycans used in the simulations. We then generated multiple repeats in conditions mirroring physiological conditions and tracked the native movement of both the WT and plasmin clipped forms to focus on the process by which the proteins transition from a compact O-shape to the extended linear forms validated in literature.

Our simulations provide a potential explanation for the increased antibody reactivity in the J-shape relative to the O-shape. Further to this, we observed the protein transitioning from the O-shape to the J- and S-shapes over time, reaching endpoints similar to published structures. The expected increase in the solvent accessible surface area (SASA) of the R39-R43 region was observed, as well as nearby regions of interest, notably T50-N56 (Iverson, 2002) and R63-F67 (the inter-domain linker between DI and DII) (Ioannou, 2007). Other nearby residues were observed to have significant shifts, both spatially and in their resulting SASA, which may stabilise the epitope region in the J- and S-shapes potentially influencing antibody binding. The same changes were observed and exacerbated in simulations of the clipped β2GPI starting from the same position suggesting that whilst plasmin cleavage does not necessarily force the opening of the protein, it may influence the speed with which it transitions to different structures, the pathway to the final state, and the relative population distribution of these structures. All of these results reveal the mechanism by which β2GPI opens, and the underlying causes of the experimentally observed increased antibody response in APS patients due to the increased ratio of clipped protein.

## Methods

### Preparing molecular variants of β2GPI for molecular dynamics simulations (MDS)

**Fig. 2:**
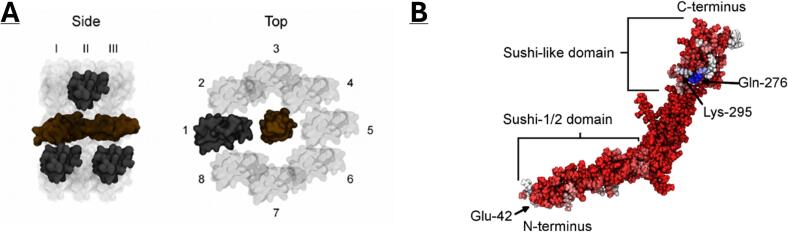
**A:** Orientation for the affinity analysis of β2GPI DI-DII (ochre) vs DV (grey). A total of 3 positions were selected at 8 different angles to give a total of 24 orientations. **B:** Affinity analysis plotted across the protein with low probability in red, medium probability in white and high probability in blue. The highest interactions are seen for Q276, however, specificity for binding was highest for K295.

A theorised circular model of β2GPI was generated starting from the linear crystal structure (PDBID: 1C1Z) without glycans (Schwarzenbacher, 1999). From experimental literature data, it is not clear where exactly the ring closes, and a head-to-head closure between DI and DV or a closure between DV and the DI-II interface are plausible. To investigate the contact point, an affinity analysis was run between DI-II and DV using MDS. A total of 24 positions were generated by rotating DV around the main axis of DI-DII at three distinct positions relative to the DI-II axis (Fig. 2). The system was minimised, solvated, and equilibrated for 20 ps in a canonical ensemble (NVT) and 50 ps in an isothermal-isobaric (NPT) ensemble. Close contact probabilities between domains were then calculated for 70 ns by counting the occurrences of distances below 0.24 nm between all atoms. The most specific interaction was seen between K295 and E42, and ring closure was assumed to occur at this contact point. To close the ring, a flat-bottom potential was applied between the amino group nitrogen of K295 and the carboxyl group carbon of E42 in a 70 ns simulation. The force constant of the flat-bottom potential was 1,000 kJ/mol/nm^2^, with a lower and upper wall at 0.15 and 0.4 nm, respectively. This closed ring structure was not stable after removing the restraint, but the ring could be further stabilized by the inclusion of branched glycans as described by Kondo *et al* (Kondo, 2009). This theorised circular model acted as the basis for subsequent simulations. Protonatable residues were edited on CHARMM-GUI for correct ionisation at pH 7.

Plasmin cleavage was based on the high-resolution (2.86 Å) X-ray crystal structure of β2GPI (PDB ID: 1C1Z) (Schwarzenbacher, 1999). To model the plasmin cleaved variant, amino acids 318–326 (the final 8 N-terminal amino acids) were deleted, as has been suggested to take place experimentally (Bradford, 2025). Both variants had four biantennary sialylated glycans (Man_3_GlcNAc_2_ core and two NeuNAc.Gal.GlcNAc antennae) attached to N143, N164, N174, N234 as detected by Kondo et al (Kondo, 2009) and Baerenfaenger (Baerenfaenger and Meyer, 2019).

### Molecular dynamics

Ring opening trajectories were simulated using the nanoscale molecular dynamics 2 (NAMD2) program (Phillips, 2020). The simulation conditions were set using the CHARMM-GUI (Jo et al., 2008, Brooks, 2009): temperature 303.15 K, pH 7, ionic strength 0.15 M simulated with NaCl ions, pressure 1 bar, in explicit solvent with TIP3P water molecules (MacKerell, 1998) and counter-ions to neutralise charge in the simulation box. The box was set to leave at least 1 nm distance from the protein on each axis. The CHARMM36 forcefield was used (MacKerell, 1998, Huang and Mackerell, 2013, Mackerell et al., 2004). The particle mesh Ewald algorithm with a spacing of 0.1 nm was applied to calculate long-range electrostatic forces (with the neighbour list updated every 40 fs). The short-range electrostatic and Van-der-Waals interactions were calculated with a cutoff of 1.2 nm. The Van-der-Waals interactions were smoothly switched off at 1 nm by a force-switching function (Steinbach and Brooks, 1994). SHAKE was used to constrain all bonds involving hydrogen atoms. The timestep used was 2 fs, with coordinates saved every 100 ps. Both systems, wildtype and plasmin-clipped β2GPI, were equilibrated in NVT (Phillips, 2020). Positional restraints for carbon alpha backbone atoms were applied to ensure gradual equilibration of the system over 100,000 steps (200 ps). Simulations were run in NPT for a total of 150 ns, with the Langevin coupling coefficient set to 1 ps^−1^ and a Nose-Hoover Langevin piston was used to maintain constant pressure (Martyna et al., 1994, Feller et al., 1995); with a piston period of 50 fs and a piston decay of 25 fs.

### Path similarity analysis (PSA)

PSA was measured with the MDAnalysis python package (Gowers, 2016, Michaud-Agrawal et al., 2011, Seyler et al., 2015) on the 10 repeats of the WT model and the 10 repeats of the clipped model separately, then plotted on a common scale to compare the relative similarity of both models to their respective repeats (Venanzi, 2024). The Hausdorff method was used, which measures the root mean square deviation (RMSD) between pairs of conformations in two trajectories. The final value is the RMSD between one frame from one trajectory and its least similar nearest neighbour in the other trajectory. It follows that higher values indicates increasingly dissimilar trajectories.

$$\delta_{H}\left(P|Q\right)=\max_{p\in P}\min_{q\in Q}d\left(p,q\right)$$

Where $\delta_{H}\left(P|Q\right)$ is the Hausdorff distance between two trajectories *P* & *Q*, *p* & *q* are frames within the trajectories *P* & *Q*, respectively.

### Principal component analysis (PCA)

Coordinate PCA was performed on a combined trajectory of the 10 repeats of WT and 10 repeats of clipped models of β2GPI. This was achieved with the R library Bio3D (Grant et al., 2006), using the “average” algorithm for hierarchical clustering, using the first 4 principal components and splitting the frames into 5 clusters in the PCA dimensional space. By combining all the simulations into one single trajectory and performing PCA and clustering on this combined trajectory, we ensure the principal components used for the clustering are the same for every repeat and model and represent the composite PC weighting that best represents all the motions identified in the full dataset. This can then provide information about the distribution of the structural clusters as a function of the model and condition used in each simulation (Lalaurie, 2022, Lalaurie et al., 2024, Spiteri, 2021, Spiteri, 2021).

### Root mean square deviation (RMSD), Solvent-accessible surface area (SASA) and root mean square Fluctuation (RMSF) Measurements

RMSD, SASA and RMSF were measured using the visual molecular dynamics (VMD) software (Humphrey et al., 1996) and tool command language (TCL) scripts to iterate over the frames and residues. RMSD was measured for the full protein, after aligning the full protein, and using a nested loop to use each frame as a reference to measure the RMSD from. RMSD is used to provide information on structural similarities between two frames, with low RMSD values indicating similar conformations and high RMSD values indicating dissimilar structures, though the same high-value RMSD can be due to two different structures owing to the averaging over the whole structure (Chen and Deng, 2007). It is therefore important to supplement this information with PCA and clustering to identify correct conformational groups. SASA was measured for every residue over the trajectory and was used to identify residues which are exposed to the solvent. This can aid in identifying potential epitope regions. This can be measured for the whole protein and for each residue individually, giving insight into the behaviour of specific peptides within the structure by monitoring the SASA over time (Palm, 1998). RMSF was measured over 25 ns fragments of each trajectory. RMSF was used to measure the displacement of each residue around its average position within the timeframe studied. Here we split the trajectories into six 25 ns fragments and measured the RMSF of each residue within that timeframe. This provided information on the gradual stabilisation, or increased flexibility, of residues within the protein (Al Qaraghuli et al., 2018, Badshah et al., 2016).

### Dynamic cross correlation (DCC) analysis

DCC matrices (DCCMs) were generated with the Bio3D package in R (Grant et al., 2006). These provide information on the simultaneous movements of residues across the protein. This helps in identifying short peptides that can affect other residues’ movement despite being distant in the sequence, and sometimes spatially (Kellerman et al., 2022).

## Results and discussion

### PSA and RMSD measurements reveal increased repeatability in the plasmin clipped model

**Fig. 3:**
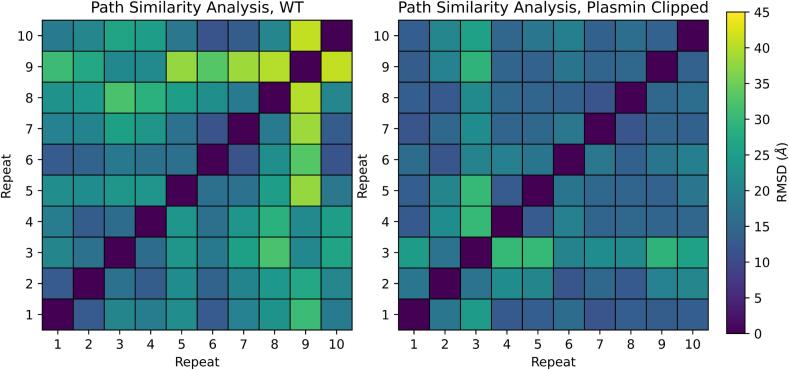
Path similarity analysis of the ten repeats of WT (left) and clipped (right) models, revealing a more common pathway across the ten repeats of the clipped relative to the WT model.

Starting from an identical (excluding the cleaved peptide) theoretical model of the O-shape of β2GPI, ten repeats of the protein were simulated for 150 ns for both the WT and the clipped β2GPI models. Initial analysis by PSA (Fig. 3) revealed a higher repeatability within the plasmin clipped trajectories when compared to the WT model. Therefore, within each models’ conformational space, the clipped protein repeatedly found itself in the same conformation more often than the WT model. This increased similarity between repeats of the clipped model was further confirmed by a more detailed pairwise RMSD analysis of each frame to every other frame in the repeats (Supplementary Fig. S2). This confirmed that every repeat explored common conformations, as indicated by the low RMSD values after aligning the full protein. In addition, the low RMSD values were often found along the diagonal, meaning the protein explored common conformations at similar timepoints within the simulations. This suggests that the simulations are robust, as they repeatedly explore the same conformations at similar times. Supplementary Fig. S3 shows that the RMSD (A, WT & B, plasmin clipped) and R_G_ (C, WT & D, plasmin clipped) values have plateaued for the final 50 ns of each repeat, confirming that the simulations have reached their final state within each repeat.

### PCA identifies the classical shapes of β2GPI and highlights a differential distribution of shapes between WT and plasmin clipped models

**Fig. 4:**
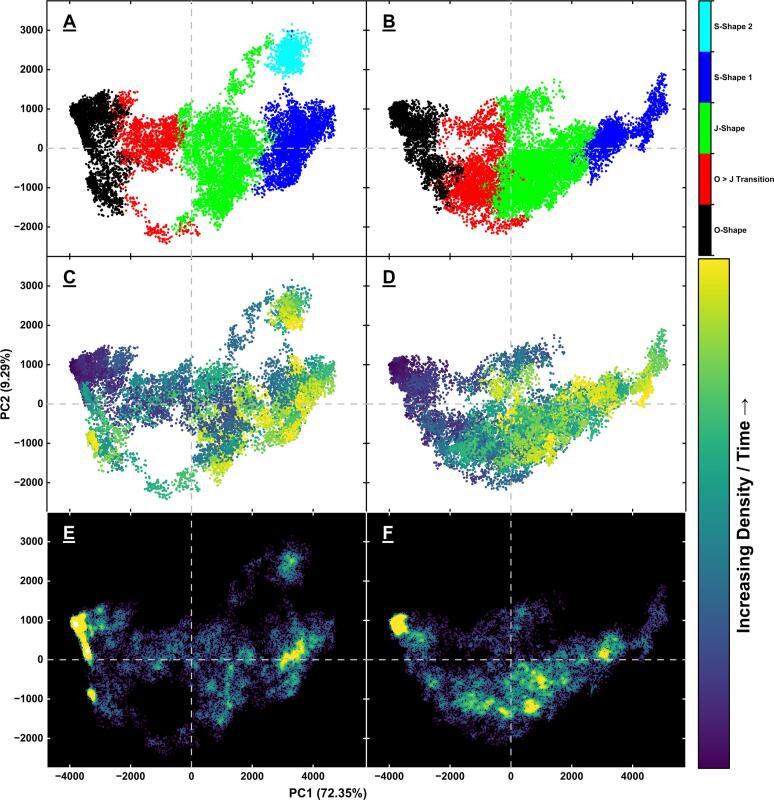
PC1 vs PC2 plots of the WT (**A, C, E**) and clipped (**B, D, F**) models, coloured by cluster (**A, B**), time within each repeat (**C, D**) and local density of points (**E, F**). This reveals a differential pathway from the O-shape to the J-shape (**A, B**), and a difference in the end-points of the simulations (**C, D**) and in the population distribution between the clusters (**E, F**) in the clipped relative to the WT models.

**Fig. 5:**
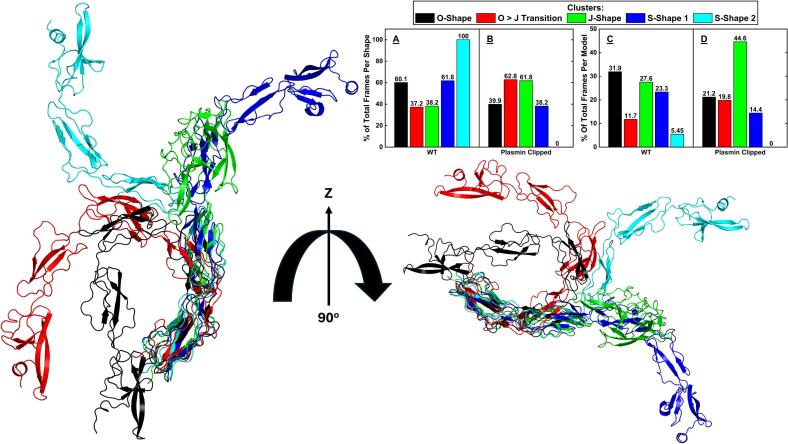
Cartoon representation of the cluster mid-points showing the O, transition, J and S-shapes. **A, B:** Population distribution of the shapes by model, showing the increased representation of the O- and S-shapes in the WT, and the increased representation of the transition and J-shapes in the clipped. Sum for each shape is 100%. **C, D:** Population distribution of the shapes within each model, showing the increased proportion of frames in the O- and S-shapes for the WT, and the increased proportion of frames in the transition and J-shape in the clipped. Sum for each model is 100%.

**Fig. 6:**
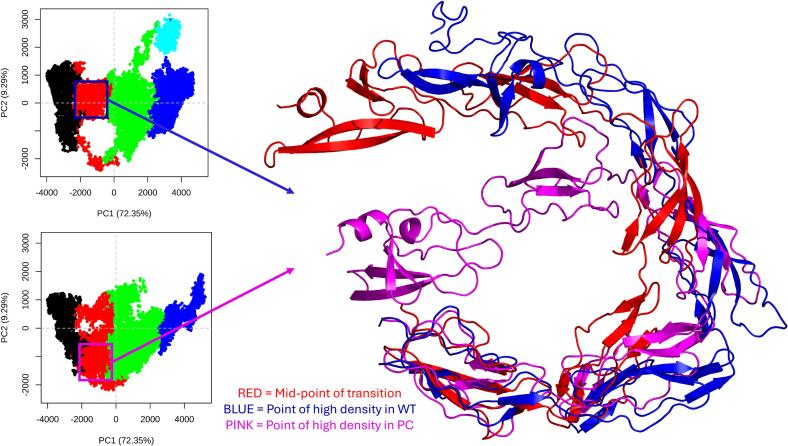
Cartoon representation of the different pathways from O-shape to J-shape in the WT (blue) and clipped (pink) models. In red is the mid-point of that structural cluster when combining both models. The difference happens at the hinge linking DII to DIII, with DV in the WT moving “upwards” while in the clipped it first moves “outwards” from DI.

While RMSD is a good measure of conformational similarity, it does not provide information on the range of structures accessed within a simulation. To better characterise the distribution of structures within the simulations we therefore conducted principal component analysis (PCA) on a compiled trajectory containing all 10 repeats of the WT and the clipped models and used this as the basis for hierarchical clustering of the frames into groups of common conformations. The results confirmed that grouping into five major conformations was sufficient and that using the first four principal components covered 89.3 % of the total variance within (Supplementary Fig. S4). Plotting the data for PC1 vs PC2, of which the motions are represented in Supplementary Fig. S5), and colouring by cluster (Fig. 4A, B), confirmed the separation of these clusters in PC space, validating they were distinct enough to warrant using five clusters, as suggested by the elbow plot. The five conformations identified were a good match to previously identified and published structures of β2GPI, with the starting O-shape (Aǧar, 2010), a transition state as the protein moves from the O-shape to the J-shape, the J-shape itself (Schwarzenbacher, 1999), and two distinct S-shapes (De Groot and Meijers, 2011) (Fig. 5). The identification of these structures, the RMSD relative to the starting point reaching a plateau, and the absence of experimental evidence showing that β2GPI is able to re-circularise spontaneously at physiological conditions all suggests the simulations have come to a point of equilibrium. It also highlights that the simulations have a high degree of accuracy, as they have spontaneously, and without constraint, reached well established conformations of β2GPI despite starting from a theoretical, unconfirmed model. This further revealed that the WT model and the clipped model transitioned from the O-shape to the J-shape through different pathways, with the main difference seen at the DII-DIII hinge (Fig. 6). Colouring the data points by time in the simulation (Fig. 4C, D) revealed another difference, with the WT staying within the O-shape for longer periods of time on average, and preferentially exploring the two S-shapes, whereas the clipped form favoured the J-shape. This was further confirmed by measuring the local density of points (Fig. 4E, F) which highlighted an increase in density within the O- and S-shapes for the WT relative to the clipped, and an increased density within the J-shape for the clipped relative to the WT.

The distribution of the shapes between the WT and clipped models was a first indication of the increased exposure of the antigenic site for the clipped protein. The WT model accounted for 60.1 % of the O-shapes across both simulations while the clipped model accounted for 39.9 % (Fig. 5A, B) suggesting a propensity for the clipped model to open faster. Looking at each model separately, the O-shape represented 31.9 % of the total frames of the WT but just 21.2 % of the clipped (Fig. 5C, D) suggesting that even within each simulation the WT remained in the O-shape for longer.

In contrast, the WT model accounted for just 37.2 % and 38.2 % of the total frames in the O to J transition and J-shapes, respectively, while the clipped model accounted for 62.8 % and 61.8 % of these clusters, respectively. This suggests an increased conformational flexibility, or an increased inherent instability of the O-shape, in the clipped model relative to the WT. Within each model, the O to J transition and the J-shapes represented just 11.7 % and 27.6 % of total frames in the WT model, but 19.8 % and 44.6 % of total frames in the clipped model, respectively.

Interestingly, the first S-shape (blue) was dominated by the WT model and the second S-shape (cyan) was exclusive to it. The overall increase of population in the transition and J-shapes for the clipped model hinted at a potential for increased antibody binding, linked to the increased antibody binding potential of those two conformations (De Laat et al., 2006, De Laat, 2011). The general path was as follows: simulations started in the O-shape, moved into the transition state, then to the J-shape, and from this J-shape went in either one of two directions to form the S-shape 1 and S-shape 2 clusters, though the latter was exclusive to a single repeat of the WT model. The WT model occasionally moved between the O- and transition shapes in both directions; while the clipped model, once out of the O-shape kept moving further from it highlighting an increase in the inherent instability of the O-shape in this model relative to the WT. It was also observed that the clipped model spent less time in the initial O-shape and reached its final conformation faster than the WT model, as evidenced by a K-means convergence analysis using 5 clusters and a rolling 10 frame window (Supplementary Fig. S6).

These findings suggest that there are unique structures the plasmin clipped β2GPI does not explore or would only do so rarely such that we have not observed this in the work presented here. And conversely, the clipped protein explores certain shapes as it transitions from the O- to the J-shape which the WT does not. The results seen with these simulations therefore appear to indicate that the effect of the cleavage is to speed up the opening, modify the route from the O- to the J-shape, and change the population distribution between the J- and S- shapes in favour of the S-shapes for WT and in favour of the J-shape for the clipped protein.

### SASA analysis identifies a concerted action of multiple regions to act as a discontinuous epitope across DI & DII

**Fig. 7:**
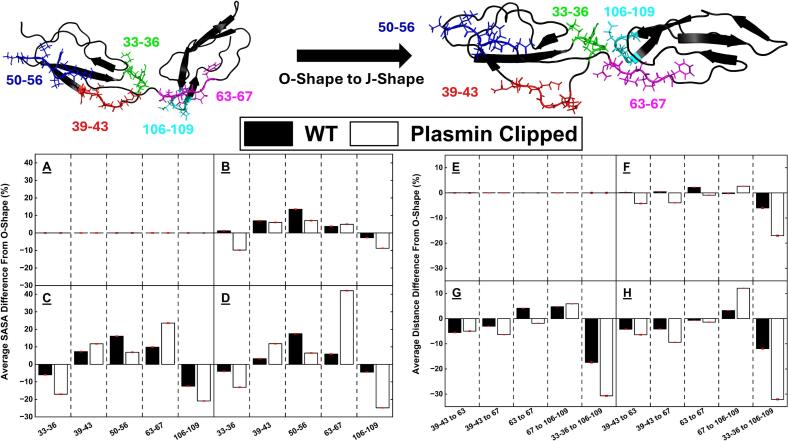
**A-D:** SASA difference from the O-shape average for regions of interest in the O-shape (**A**), transition state (**B**), J-shape (**C**) and S-shape 1 (**D**) for the WT and the clipped models. **E-H:** Distance difference from the O-shape average for regions of interest in the O-shape (**E**), transition state (**F**), J-shape (**G**) and S-shape 1 (**H**) for the WT and the clipped models. Data for the S-shape 2 is not shown here as it was exclusive to the WT, SASA stats for all shapes are shown in Table 1, Table 2, Table 3, Table 4, Table 5.

Literature suggests the dominant pathogenic epitope, R39-R43, has greater exposure in the J-shape relative to the O-shape (McDonnell, 2020, De Laat, 2011), we therefore took the opportunity to analyse the epitope’s exposure during opening. The SASA data for regions of interest is summarised in Table 1, Table 2, Table 3, Table 4, Table 5 as a percentage difference from the O-shape average within each model, and as a difference from the same shape in the clipped model relative to the WT. Fig. 7A-D shows the percentage difference of these regions of interest relative to the O-shape average within each model, alongside a visual representation of these regions in the O- and J-Shapes.

**Table 1:** SASA difference of the 33–36 peptide in different shapes between WT and clipped models.

	**WT**	**Clipped**	
**% Difference from …**	**O-shape (WT)**	**O-shape (Clipped)**	**Same shape (WT)**
**O-shape**	N/A		+0.13 ± 0.22 %
**O > J Transition**	+1.24 ± 0.29 %	−9.78 ± 0.24 %	−10.77 ± 0.24 %
**J-shape**	−5.94 ± 0.18 %	−17.04 ± 0.11 %	−11.69 ± 0.12 %
**S-shape 1**	−3.98 ± 0.19 %	−13.06 ± 0.15 %	−9.34 ± 0.15 %
**S-shape 2**	+14.50 ± 0.24 %	N/A

**Table 2:** SASA difference of 39–43 peptide in different shapes between WT and clipped models.

	**WT**	**Clipped**	
**% Difference from …**	**O-shape (WT)**	**O-shape (Clipped)**	**Same shape (WT)**
**O-shape**	N/A		+4.42 ± 0.23 %
**O > J Transition**	+6.97 ± 0.36 %	+6.00 ± 0.31 %	+3.48 ± 0.30 %
**J-shape**	+7.29 ± 0.19 %	+11.75 ± 0.19 %	+8.76 ± 0.18 %
**S-shape 1**	+3.22 ± 0.29 %	+11.80 ± 0.30 %	+13.11 ± 0.30 %
**S-shape 2**	+15.96 ± 0.40 %	N/A

**Table 3:** SASA difference of 50–56 peptide in different shapes between WT and clipped models.

	**WT**	**Clipped**	
**% Difference from …**	**O-shape (WT)**	**O-shape (Clipped)**	**Same shape (WT)**
**O-shape**	N/A		+12.92 ± 0.27 %
**O > J Transition**	+13.54 ± 0.27 %	+7.05 ± 0.18 %	+6.47 ± 0.18 %
**J-shape**	+16.06 ± 0.17 %	+6.88 ± 0.11 %	+3.99 ± 0.11 %
**S-shape 1**	+17.47 ± 0.17 %	+6.45 ± 0.15 %	+2.32 ± 0.15 %
**S-shape 2**	+27.54 ± 0.25 %	N/A

**Table 4:** SASA difference of 63–67 peptide in different shapes between WT and clipped models.

	**WT**	**Clipped**	
**% Difference from …**	**O-shape (WT)**	**O-shape (Clipped)**	**Same shape (WT)**
**O-shape**	N/A		+1.43 ± 0.25 %
**O > J Transition**	+3.75 ± 0.50 %	+4.95 ± 0.35 %	+2.60 ± 0.34 %
**J-shape**	+9.81 ± 0.36 %	+23.53 ± 0.27 %	+14.10 ± 0.25 %
**S-shape 1**	+5.85 ± 0.39 %	+42.05 ± 0.29 %	+36.12 ± 0.28 %
**S-shape 2**	−21.70 ± 0.29 %	N/A

**Table 5:** SASA difference of 106–109 peptide in different shapes between WT and clipped models.

	**WT**	**Clipped**	
**% Difference from …**	**O-shape (WT)**	**O-shape (Clipped)**	**Same shape (WT)**
**O-shape**	N/A		+6.38 ± 0.17 %
**O > J Transition**	−2.70 ± 0.29 %	−8.75 ± 0.25 %	−0.23 ± 0.27 %
**J-shape**	−12.45 ± 0.21 %	−20.90 ± 0.13 %	−3.89 ± 0.16 %
**S-shape 1**	−4.40 ± 0.23 %	−24.82 ± 0.18 %	−16.35 ± 0.20 %
**S-shape 2**	−1.61 ± 0.33 %	N/A

While some residues are widely recognised as being the main contributors to the epitope, doubt remains as to its exact nature. Through carrying out a SASA analysis of residues within DI, and at the interface between DI and DII, we identified several residues which we suggest may play a part in a discontinuous epitope on the surface of DI and DII. Analysis of residues R39-R43 revealed there was an increase in SASA for that peptide relative to the O-shape in all of the other shapes, with a larger increase in the clipped model. This reflects data in literature suggesting that plasmin clipped β2GPI binds autoantibodies with greater efficiency (Bradford, 2025). Even within the O-shape, the average SASA of this region for the clipped model was larger than the WT model. A closer look revealed that the specific SASA of residues R39 and R43 were increased, while that of residue M42 was decreased. This suggests that the epitope may be more complex than just this peptide, and surrounding residues may affect their local conformation and exposure levels.

The T50-N56 peptide, which was initially covered by DV in the O-shape, was left significantly more exposed in all the other shapes. The total SASA was again greater in the clipped model relative to the WT, despite a lower percentage increase from the O-shape due to its greater exposure in the closed model. Previous work by Iverson et al (Iverson, 2002) found that point mutations in this region had a negative impact on antibody binding. Similarly, the inter-domain linker region which has been identified for potential epitope binding, R63-F67, had an increased SASA as β2GPI opens from the O-shape, with a larger increase in the clipped model. However, this peptide was left with a lower SASA in the S-shape 2, which was exclusive to the WT model, suggesting that this S-shape is less available for antibody binding. This was driven largely by residues R63 and F67; and the simultaneous reduced SASA of residue 68. The DI-DII interlinker was identified by Ioannou et al (Ioannou, 2007), specifically the peptide T61-F67, as having a large impact on antibody binding with increased binding of DI plus this peptide relative to DI alone. Finally, two loops on opposing faces of DI and DII were found to be significantly more buried in the more open shapes relative to the O-shape: K33-Y36, T106-G109. This was however also different in the S-shape 2, which was only observed in the WT model, and showed an increased exposure of this region relative to the O-shape. Supplementary Fig. S7A, B shows the linear average SASA difference from the starting point for the WT and clipped models; and confirms the larger effect within the plasmin clipped model.

### Inter-domain distances show increased proximity of key peptides in the J-shape and in the plasmin clipped model relative to the WT

**Fig. 8:**
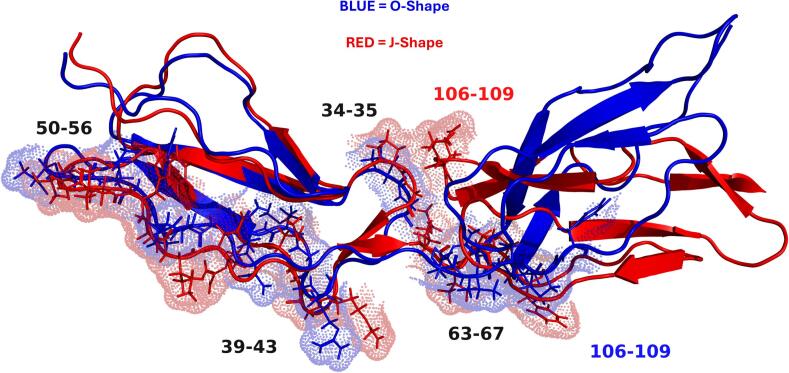
Cartoon representation of the difference of position for the peptides of interest (33–36, 39–43, 50–56, 63–67 and 106–109 in the O-shape (blue) and the J-shape (red). This shows the decrease in SASA for the 33–36, 106–109 loops and the increase in SASA for the 39–43, 50–56 and 63–67 peptides.

The observed decrease in SASA for the K33-Y36 and T106-G109 loops was identified as being driven by a movement of the T106-G109 loop towards the K33-Y36 loop due to the twisting of DII (Fig. 7E-H). This effect was larger in the clipped model, with the distance between these two loops reduced by a larger amount relative to the WT protein. Furthermore, the distance between the well-established R39-R43 epitope and the suggested DI-DII interlinker epitope was reduced in the open shapes, with this distance being the lowest in the J- and S-shape 1, specifically of the plasmin clipped protein. The increase in SASA of the R63-F67 region was largely driven by the same twisting of DII which was confirmed by the increased distance between residue F67 and the T106-G109 loop. Supplementary Fig. S7C, D shows the linear average distance difference from the starting point for the WT and clipped models, highlighting the increased effect within the clipped model. The net result of these combined changes was an increased exposure of specifically residues R39 and R43, the full peptide T50-N56 and the R63-F67 interlinker, with an overall greater increase of all these effects in the clipped model. These results are consistent with the J- and S-shape 1, in the clipped protein especially, leading to higher antibody binding relative to the O-shape by revealing an array of cryptic epitopes (R39-R43, T50-N56 and R63-F67), and bringing them closer together (R39-R43 and R63-F67). In S-shape 2 however, the R63-F67 peptide is more buried and the K33-Y36 loop is more exposed, which could be conducive to lower antibody binding ability in the WT relative to the clipped. High DCCM values between all these regions may explain the overall stabilising effect the movement of the T106-G109 loop has on the other regions (Supplementary Fig. S8). The effects mentioned here, namely the increased SASA and the reduced distances, and the movement of the T106-G109 loop are presented in Fig. 8 which highlights the peptides studied in the O- and J-Shape.

### RMSF analysis revealed a sudden stabilisation of epitope residues after two loops on DI & DII come into closer proximity

**Fig. 9:**
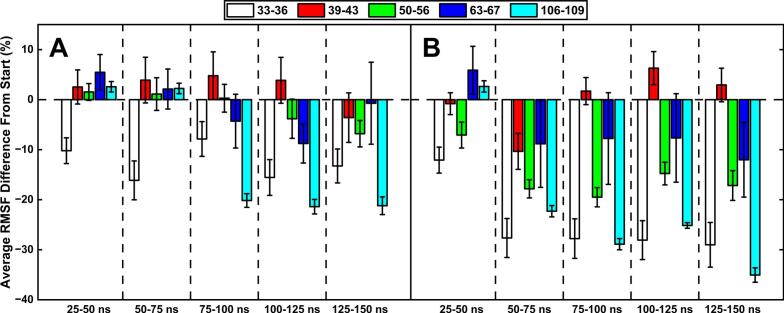
Average RMSF difference from the initial 25 ns RMSF values for regions of interest in the WT (**A**) and clipped (**B**) models. This shows the faster stabilisation of key regions in the clipped model, likely linked to the faster displacement of the 106–109 loop.

Another factor which can play a supporting role in the strength of an epitope is the local stability, or flexibility, of the residues involved. This is measured by the RMSF of residues over a given time, with a larger RMSF indicating more flexibility. Here, we have measured the average RMSF value in the first 25 ns of each simulation, and the average RMSF of every subsequent 25 ns chunks. Fig. 9 shows the percentage difference in RMSF for the subsequent 25 ns chunks relative to the initial 25 ns, which revealed that the regions of interest were more stable (i.e. negative difference relative to initial 25 ns), or very close to the starting values, in the regions likely to be linked to antibody binding (R39-R43, T50-N56, R63-F67) as the simulations went on. These regions were likely stabilised by the increased proximity of the loops K33-Y36 and T106-G109, as evidenced by the timing of the sudden increase in the WT (75–100 ns) and the clipped (50–75 ns) models, which matches the timing of the decreased/increased SASAs and distances of these regions (Supplementary Fig. S7). The slight increase in the flexibility of the clipped model’s R39-R43 region may explain the relative increase of SASA of this region compared to the WT model.

### The J-shape exposes a motif to the surface of DI & DII which is hidden in the O-shape, identified as the target for APS antibodies

**Fig. 10:**
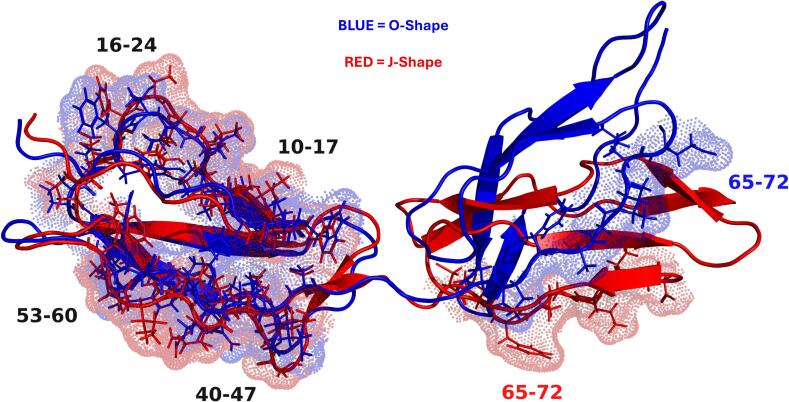
Cartoon representation of the motifs identified by de Moerloose et al (de Moerloose, 2017) on the O-shape (blue) and J-shape (red), demonstrating the increased SASA of the 40–47, 53–60 & 65–72 peptides.

**Fig. 11:**
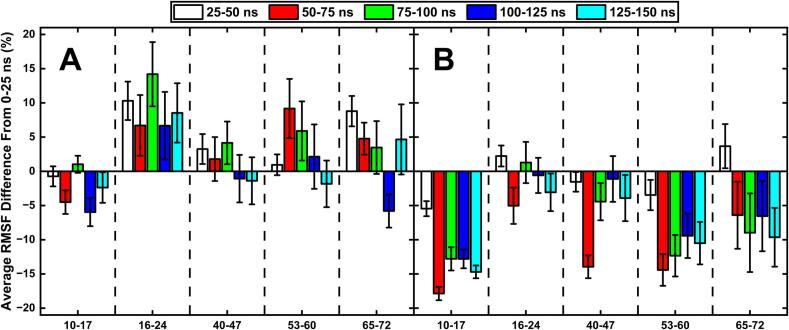
RMSF difference of the motifs present in DI & DII in the WT (**A**) and clipped (**B**) models over time; showing the increased stability in the plasmin clipped model and the increased flexibility in the WT.

Previous work by de Moerloose et al (de Moerloose, 2017) identified that the APS antibodies may recognise a pattern rather than individual residues. Their suggested patterns are ϕϕϕζζFxϕ or ϕϕϕζζFxC, with ϕ non-polar residues and ζ polar residues. As F and C are non-polar this pattern could be simplified to ϕϕϕζζϕxϕ. This pattern, or closely related ones, are present five times in DI-DII of β2GPI (Table 6). Fig. 10 represents the residues which make the motif in the O- and J-shapes. While these motifs exists regardless of the shape, their properties are greatly impacted by the conformation of the protein and the relative orientation of domains I and II. SASA analysis revealed that in the J-shape, all of these regions except 10–17 have increased exposure, by up to 9 % relative to the O-shape. In the plasmin clipped, all of these same regions have increased exposure relative to the WT, by up to 10.8 %. As well as having increased exposure, these residues displayed a greater stability in the plasmin clipped model while they displayed increased flexibility in the WT, as shown in Fig. 11.

**Table 6:** Residues and motifs identified within DI-DII of β2GPI, and their respective SASA difference from the O-shape in the J-shape; and for the clipped protein, the difference relative to the J-Shape in the WT.

		**WT**	**Plasmin clipped**	
**Residues**	**Motif**	**% SASA difference from O-shape (WT)**	**% SASA difference from O-shape**	**% SASA difference from WT J-shape**
**10**–**17**	**17-ϕϕϕζζFxϕ-10**	−3.27 ± 0.12 %	−2.62 ± 0.10 %	−1.02 ± 0.10 %
**16**–**24**	**16-ϕϕϕζζFxϕ-24**	+9.31 ± 0.08 %	+4.31 ± 0.06 %	−5.85 ± 0.05 %
**40**–**47**	**40-ϕϕϕζζFxC-47**	+1.01 ± 0.17 %	+4.24 ± 0.15 %	+10.8 ± 0.16 %
**53**–**60**	**53-ϕϕϕζζϕxC-60**	+9.03 ± 0.15 %	+3.44 ± 0.11 %	+3.74 ± 0.11 %
**65**–**72**	**72-ζϕϕϕϕFxC-65**	−1.22 ± 0.12 %	+7.37 ± 0.09 %	+4.16 ± 0.08 %

## Conclusion

Our data represents a thorough simulation of the opening of β2GPI from a compact theoretical circular form through to validated structures identified in literature. These simulations have revealed a number of interesting findings which fit contextually within the literature of β2GPI. For example, when starting from the O-shape, β2GPI opened out with DV separating from the surface of DI until it reached a J-shape, followed by one of two S-shapes. This appears to indicate a natural instability of the O-shape in solution and may explain the results obtained by Kumar et al (Kumar et al., 2023). The circular form utilised in our study is based upon identifying low energy, high affinity binding sites between the 1st and 5th domain, however, it is not experimentally validated. The stability of this closed form was majorly contributed from the large, branched glycan chains added. The observed experimental data for the circular form lacks the resolution to identify fully the binding sites, however, overlap is seen in the data of Agar et al (Aǧar, 2011) which reflects the initial overlap seen in our model.

Interestingly, the conformational change observed in this work happened on a shorter timescale in the clipped model compared to the WT model. Specific properties of regions of interest identified by published experimental work appeared to reveal a mechanism through which the J-shape may lead to increased antibody binding. Indeed, it was observed that in the J- and S-shape 1 the exposure of the R39-R43, T50-N56 and R63-F67 regions was increased. These regions have been shown to lead to decreased antibody binding when point-mutations were introduced by Iverson et al (Iverson, 2002), or increased antibody binding when the T61-F67 linker was added to DI by Ioannou et al (Ioannou, 2007). Simultaneously, the distance between the epitope R39-R43 and the R63-F67 region was reduced, as well as the distance between two loops on opposing faces of DI and DII (K33-Y36, T106-G109). This had the combined effect of increasing the SASA for the R63-F67 region, reducing the flexibility for the K33-Y36 and T106-G109 loops; and through cross-correlation effects, also reducing the flexibility of the T50-N56 and R63-F67 regions. Finally, the J-shape also presented to the surface of DI-DII a number of patterns identified by de Moelroose et al (de Moerloose, 2017) as the preferential APS antibody binding site, made up of nonpolar and polar residues (ϕϕϕζζϕxϕ) across a multitude of epitopes in domains I and II. These are initially inaccessible due to the presence of DV blocking the majority of DI. All these effects are exacerbated in the plasmin clipped model, with the SASA of all these motifs being higher than in the WT model; the distances between the K33-Y36 and T106-G109 loops and between the R39-R43 epitope and R63-F67 being lower than in the WT model; and the RMSF of K33-Y36, T50-N56, R63-F67 and T106-G109 being lower relative to the WT model. This suggests a differential autoantibody binding for the plasmin clipped protein, however, this would require further validation in laboratory studies to confirm. Some work to this effect has already been undertaken, with results aligning well with the data from the MDS presented here (Bradford, 2025). It should be noted that some data hint at the plasmin cleaved peptide (318–326) remaining bound to DV of β2GPI through a disulphide bond between C326-C288. In line with recent work showing an overall lower sized protein in SDS-PAGE (Bradford, 2025) after plasmin cleavage, we have elected to model β2GPI in the absence of the peptide. The effect of maintaining the peptide through a disulphide bond within our model is therefore unknown.

Whilst the lack of a validated circular form to begin from is a significant limitation of our study, the use of an affinity measured circular form as a starting point represents a logical compromise. Furthermore, the study focusses on the transitions from the circular form to the linear form which includes the formation of published conformations identified using biophysical approaches. This suggests that the simulation is likely accurate as it, without guide or constraint, unfolds to well established conformations. Similarly, all the potential shapes which β2GPI can theoretically adopt are not represented in this study, as we have not carried out techniques with high energy, including temperature exchange simulations, the aim of this study being to identify how the protein transitions through structures at close to physiological conditions. Such high energy methods to identify rare events would have reduced the ability to compare our findings to literature where the majority of imaging and biophysical techniques have focussed on remaining as close to physiological conditions as possible. Further to this, the inherent instability seen in the circular form would be best confirmed by free energy calculations in future simulations. It has also been shown that the reverse trajectory, i.e. from linear shape back to closed form, does not happen in physiological conditions but can be forced by extreme pH conditions (Aǧar, 2010). The relevance of attempting a recircularization simulation starting from the linear shapes is therefore limited, as it would require unrealistic conditions which would not be met *in vivo*.

The results we present in this study are the first comprehensive comparison of WT and plasmin clipped β2GPI in MDS. While they remain purely theoretical due to the *in silico* nature of the work and the unconfirmed circular starting position, the close match to previously validated experimental work holds promising outlooks for the use of MDS in future analyses of additional elusive pathogenic autoantigen epitopes. Future validation for this work is essential, including targeting the T106-G109 loop with nanobodies to investigate the impact of its movement on antibody binding; or point mutations in the R63-F67 peptide to monitor the individual impact of each residue. Additionally, mutations to the T50-N56 peptide could be used to assess the impact on β2GPI’s ability to maintain the O-shape through interactions with DV. More generally, this workflow could be applied to proteins which are known to be pathogenic, but for which experimental studies have been unable to identify the specific peptide responsible, to help narrow the scope of experimental work required.

In conclusion, MDS of β2GPI under close to physiological conditions suggest that plasmin cleavage has an effect on the unfolding of the protein from a hypothetical circular form. Our results suggest the clipped protein transitions through structures unavailable to the WT and transitions quicker towards the known linear forms. This leads to increased exposure of the antigenic domain I and increased stability of the flanking regions which may contribute to the increased antibody response seen experimentally for the plasmin clipped β2GPI. The WT on the other hand explores a conformation, unavailable to the clipped model, which has properties conducive to a reduced antibody response through the reduced exposure of a key peptide. Taken together, all these results suggest a differential antibody response in the linear J-shape relative to the O-shape, and an increased antibody response in the plasmin clipped protein relative to the WT through increased proportion of circulating J-shape.

## Author contributions

C.J.L. performed the analysis, and wrote the main manuscript. P.A.D., M.K., N.G. & M.D. reviewed and edited the manuscript. T.C.R.Mc reviewed and edited the manuscript and generated the molecular simulations.

## CRediT authorship contribution statement

**C.J. Lalaurie:** Writing – original draft, Investigation, Formal analysis, Data curation. **M. Kulke:** Writing – review & editing. **N. Geist:** Writing – review & editing. **M. Delcea:** Writing – review & editing. **P.A. Dalby:** Writing – review & editing, Supervision. **T.C.R. McDonnell:** Writing – review & editing, Supervision, Project administration, Funding acquisition, Conceptualization.

## Declaration of competing interest

The authors declare the following financial interests/personal relationships which may be considered as potential competing interests: Thomas McDonnell reports financial support was provided by Versus Arthritis. Thomas McDonnell has patent #10098960 issued to UCL BUSINESS PLC. If there are other authors, they declare that they have no known competing financial interests or personal relationships that could have appeared to influence the work reported in this paper.

## Data Availability

Data will be made available on request.

## Appendix A Supplementary material

Supplementary Fig. 1 Cartoon representation of the base model used for the simulations, including the glycans shown in blue, red, magenta and black. These are not included in the main figures for increased visibility.

Supplementary Fig. 2 Pairwise RMSD plot for ten repeats of the WT (left) and clipped (right) models. This reveals a more common pathway for the clipped models, with many of the low RMSD points found along the diagonal when comparing two trajectories. This signals common conformations at common timepoints in the simulations.

Supplementary Fig. 3 RMSD (A, B) and RG (C, D) plots of the ten repeats for the WT (A, C) and plasmin clipped (B, D) proteins, with shaded area representing 5 % of the final value. This shows the plateau reached for the final 50 ns of the simulation, confirming they are unlikely to move away from their final state.

Supplementary Fig. 4 Elbow plot (left) and Scree plot (right) of the combined trajectory used for the PCA study. This confirmed that 5 structural clusters were sufficient to capture the majority of the conformations and that using the first 4 principal components captured 89.3 % of the total variance.

Supplementary Fig. 5 Directions of the motions identified by PCs 1 (black) and 2 (red). PC1 measures the “horizontal” motion along the DI-DII axis; PC2 measures the “vertical” motion of DIV-DV relative to DI-DII.

Supplementary Fig. 6 Rate of convergence of the WT (top) and clipped (bottom) models using a K-means clustering with 5 clusters and a rolling 10 frame window. This revealed an overall faster exit from the O-shape for the clipped models and a faster convergence to the final state of their simulations compared to the WT.

Supplementary Fig. 7 Average differences from the starting point for SASA (A, B) and distance (C, D) of regions of interest in the WT (A, C) and clipped (B, D) models. This highlights the increased effects seen in the clipped models with larger differences from the starting point relative to the WT.

Supplementary Fig. 8 Dynamic cross-correlation plot for DI-DII, with regions of interest highlighted. High cross-correlation between all the regions of interest could indicate the overall stabilising effect of the 106 109 loop displacement on the main R39-R43 epitope.
