# Supplementary figures and images for: The J-shape of β2GPI reveals a cryptic discontinuous epitope across domains I and II
Source: J Struct Biol X. 2025 Aug 20;12:100135. doi: 10.1016/j.yjsbx.2025.100135 (PMC12398265; doi:10.1016/j.yjsbx.2025.100135)

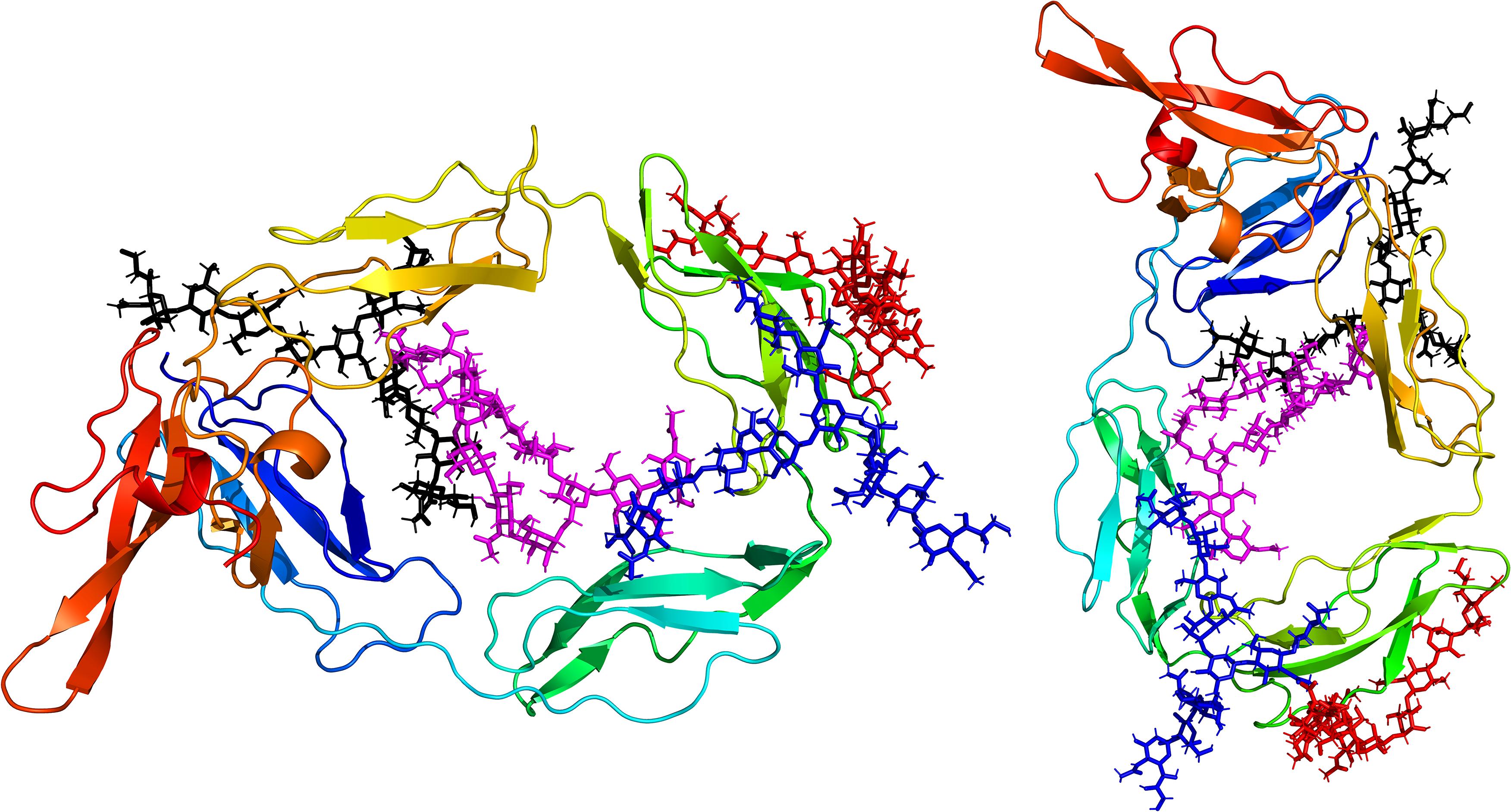

Supplement: Supplementary Fig. 1 — Cartoon representation of the base model used for the simulations, including the glycans shown in blue, red, magenta and black. These are not included in the main figures for increased visibility. [file mmc1.jpg]

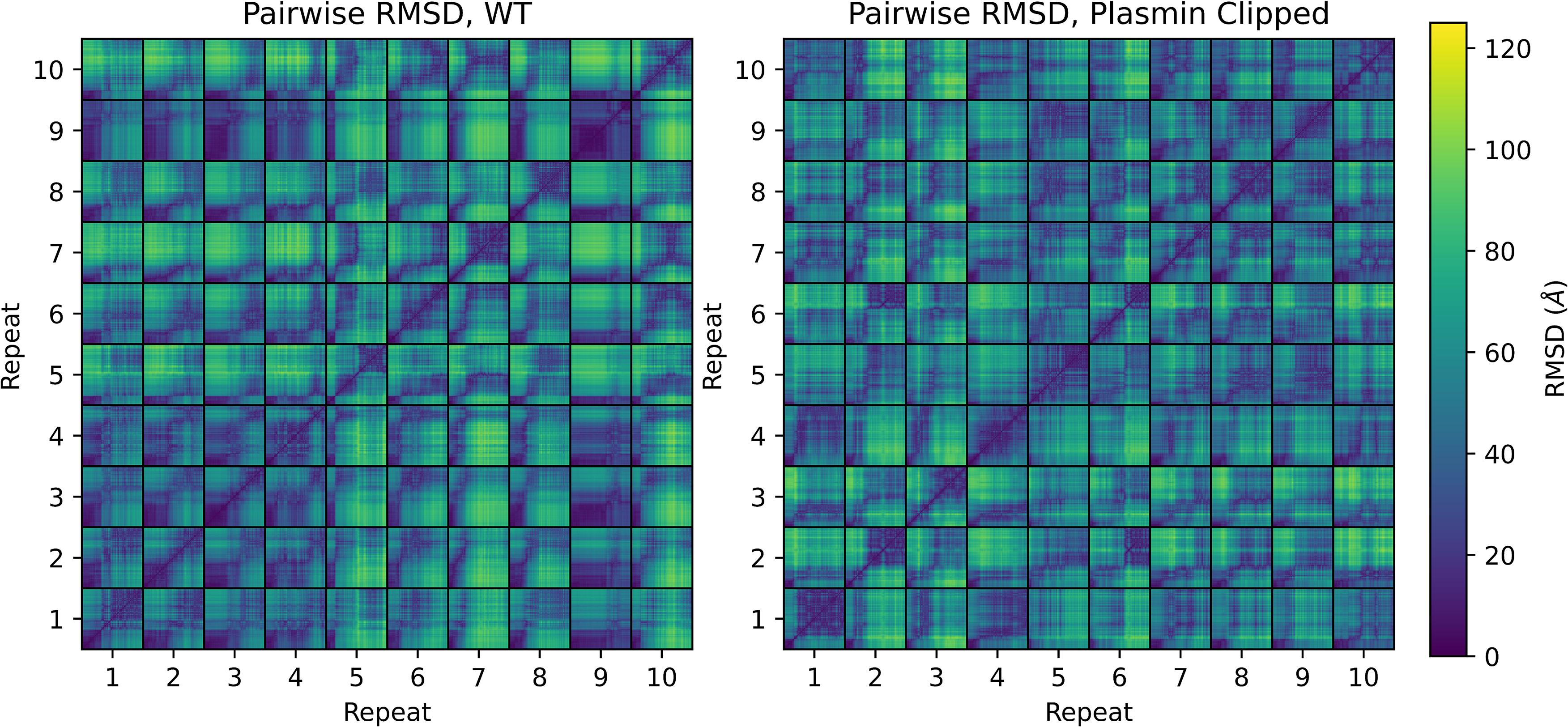

Supplement: Supplementary Fig. 2 — Pairwise RMSD plot for ten repeats of the WT (left) and clipped (right) models. This reveals a more common pathway for the clipped models, with many of the low RMSD points found along the diagonal when comparing two trajectories. This signals common conformations at common timepoints in the simulations. [file mmc2.jpg]

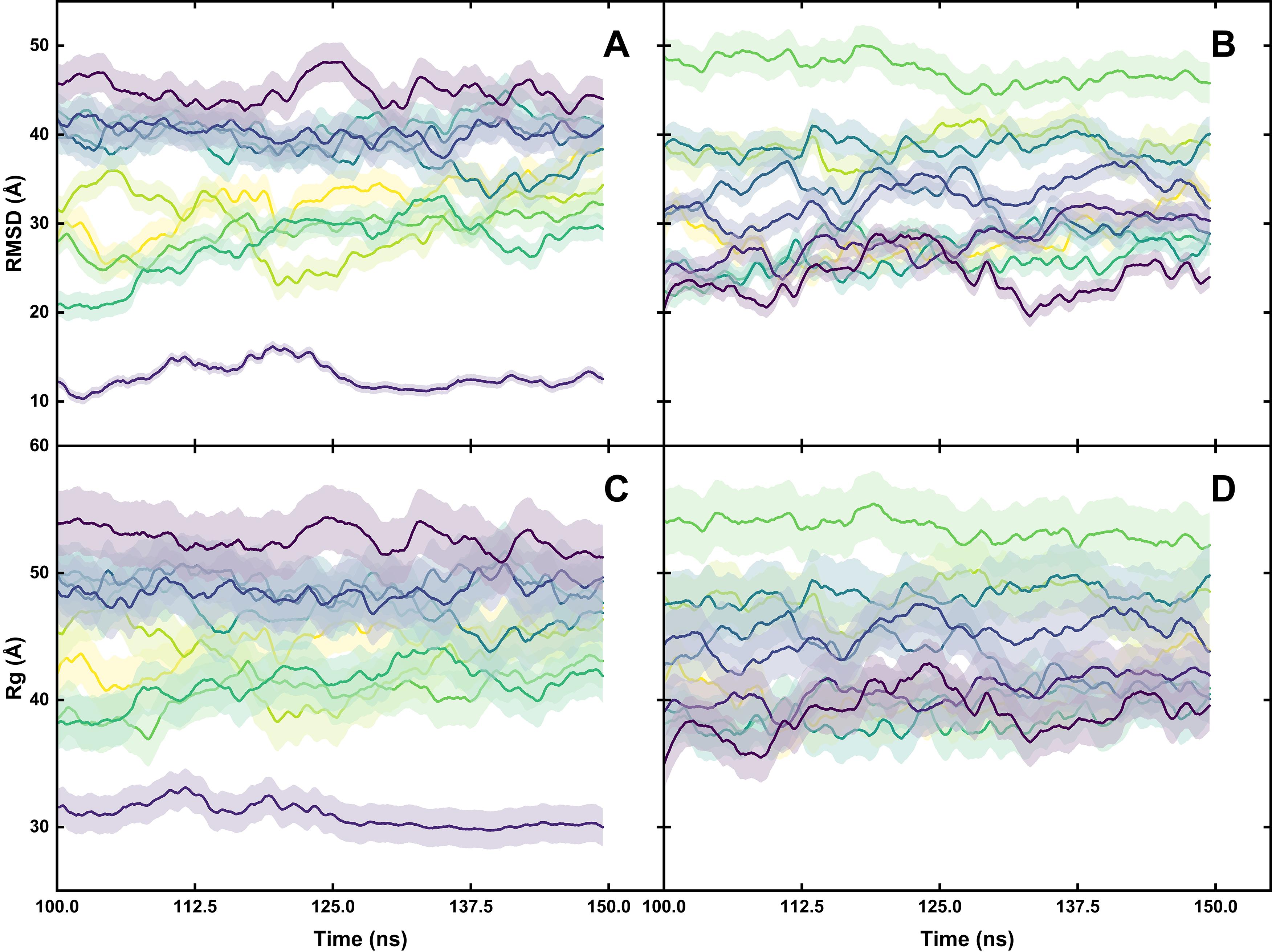

Supplement: Supplementary Fig. 3 — RMSD (A, B) and RG (C, D) plots of the ten repeats for the WT (A, C) and plasmin clipped (B, D) proteins, with shaded area representing 5 % of the final value. This shows the plateau reached for the final 50 ns of the simulation, confirming they are unlikely to move away from their final state. [file mmc3.jpg]

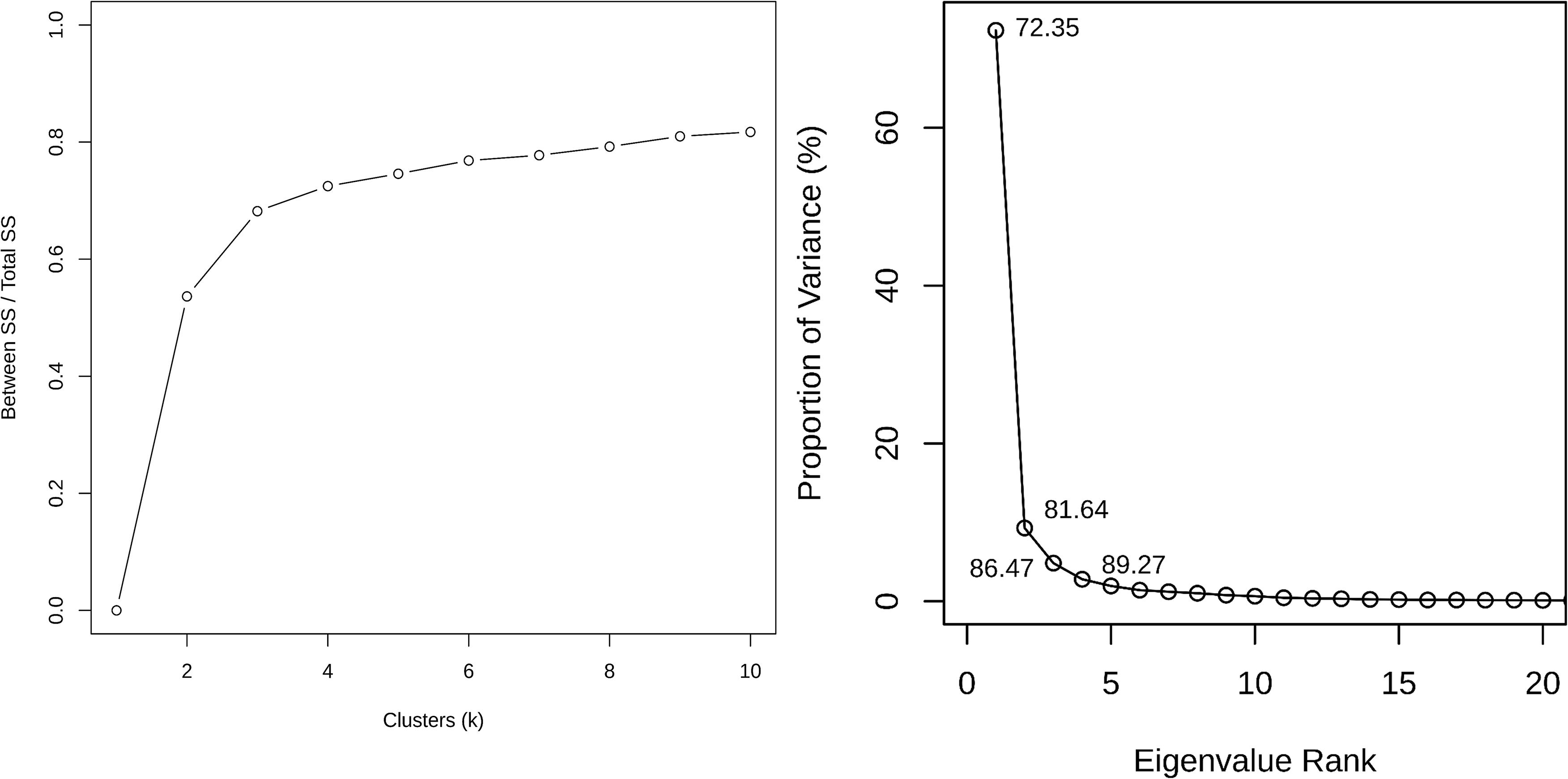

Supplement: Supplementary Fig. 4 — Elbow plot (left) and Scree plot (right) of the combined trajectory used for the PCA study. This confirmed that 5 structural clusters were sufficient to capture the majority of the conformations and that using the first 4 principal components captured 89.3 % of the total variance. [file mmc4.jpg]

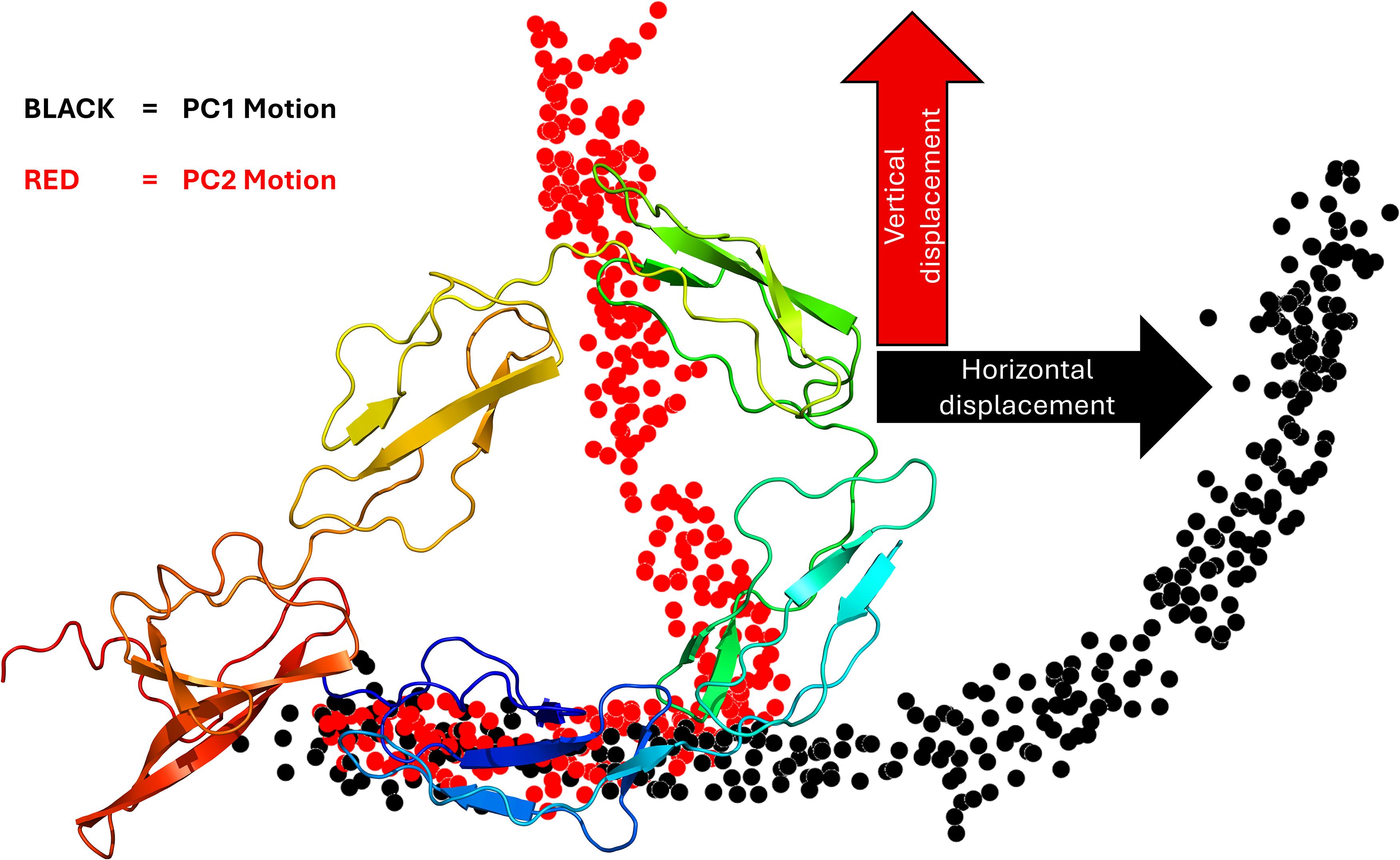

Supplement: Supplementary Fig. 5 — Directions of the motions identified by PCs 1 (black) and 2 (red). PC1 measures the “horizontal” motion along the DI-DII axis; PC2 measures the “vertical” motion of DIV-DV relative to DI-DII. [file mmc5.jpg]

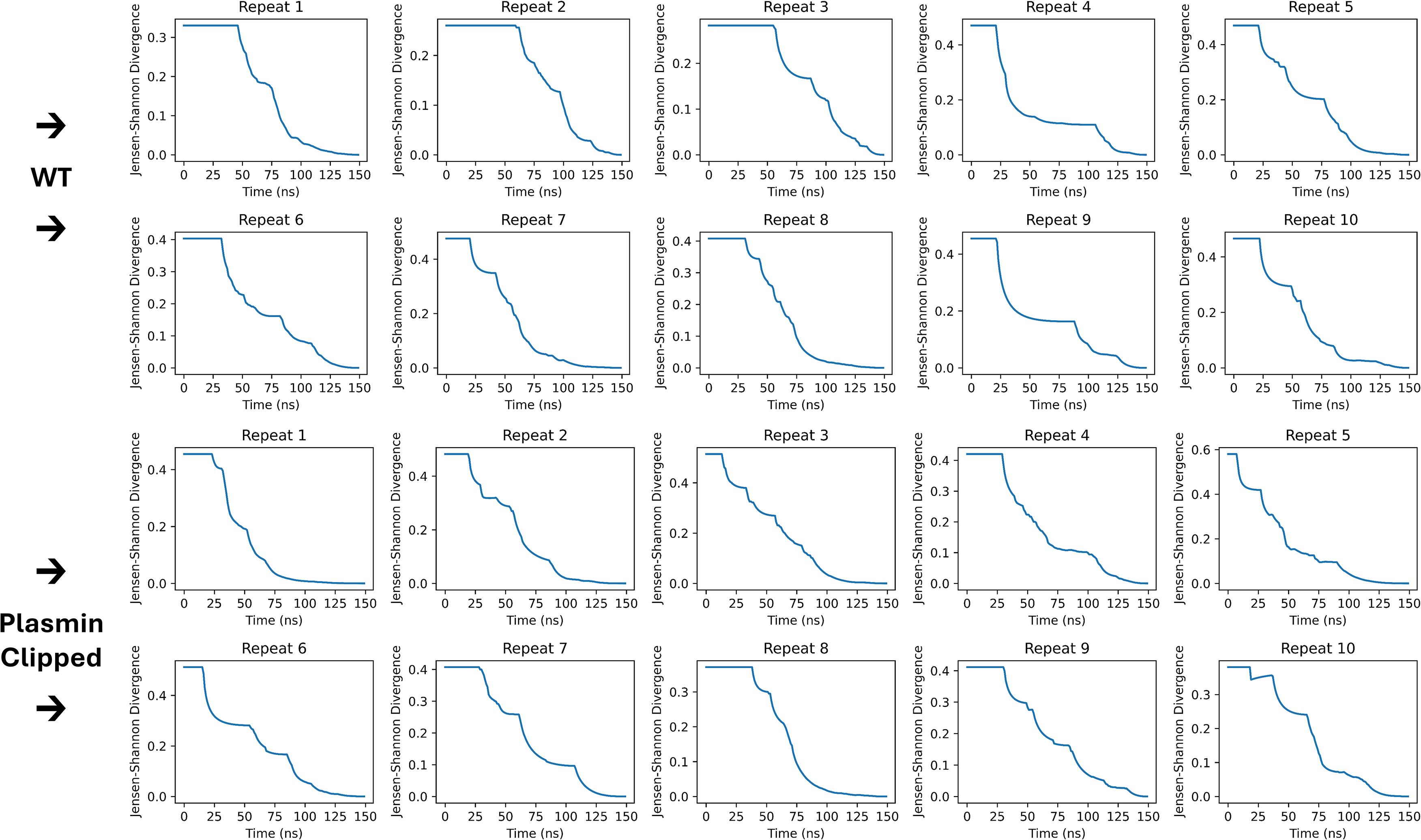

Supplement: Supplementary Fig. 6 — Rate of convergence of the WT (top) and clipped (bottom) models using a K-means clustering with 5 clusters and a rolling 10 frame window. This revealed an overall faster exit from the O-shape for the clipped models and a faster convergence to the final state of their simulations compared to the WT. [file mmc6.jpg]

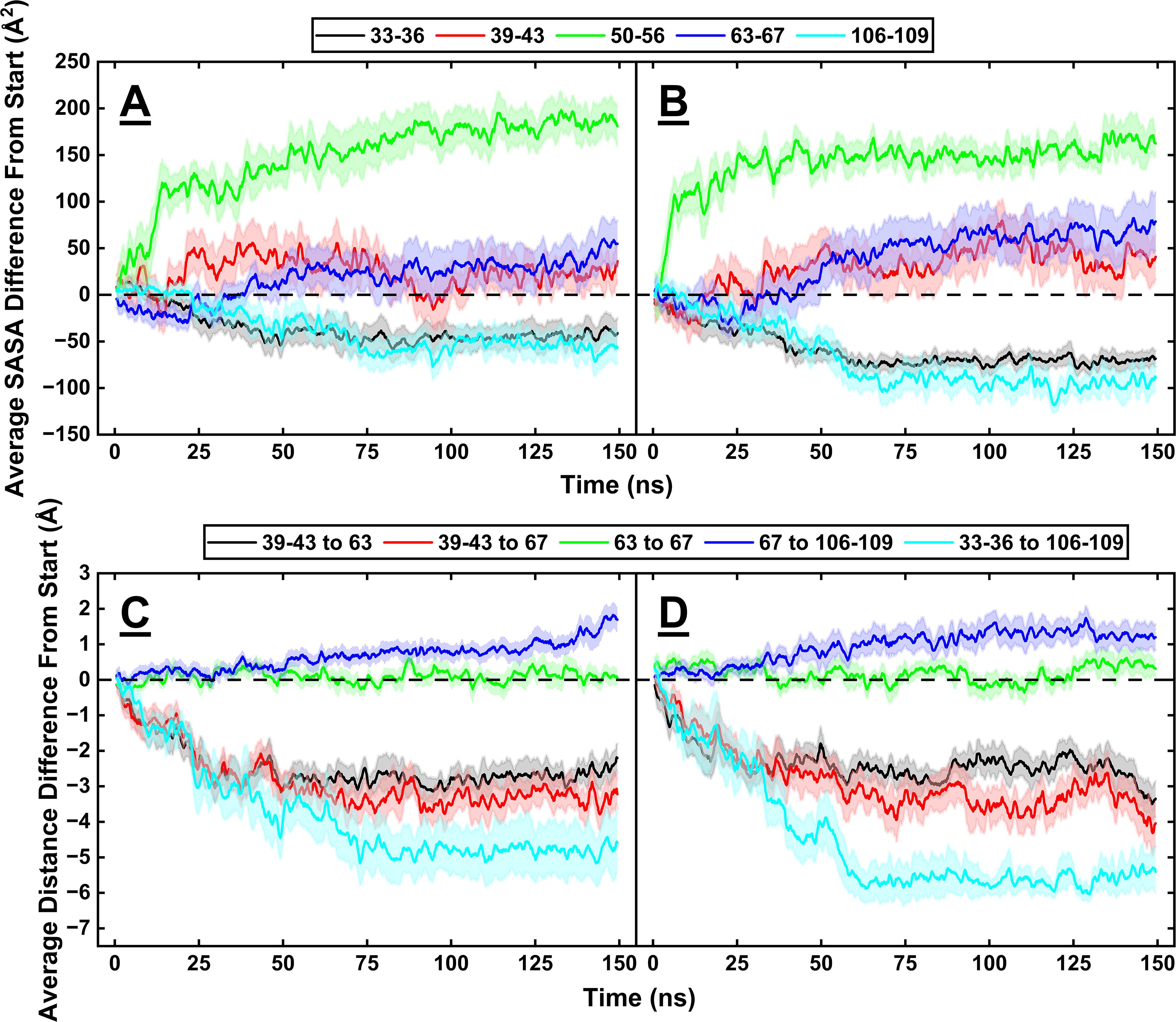

Supplement: Supplementary Fig. 7 — Average differences from the starting point for SASA (A, B) and distance (C, D) of regions of interest in the WT (A, C) and clipped (B, D) models. This highlights the increased effects seen in the clipped models with larger differences from the starting point relative to the WT. [file mmc7.jpg]

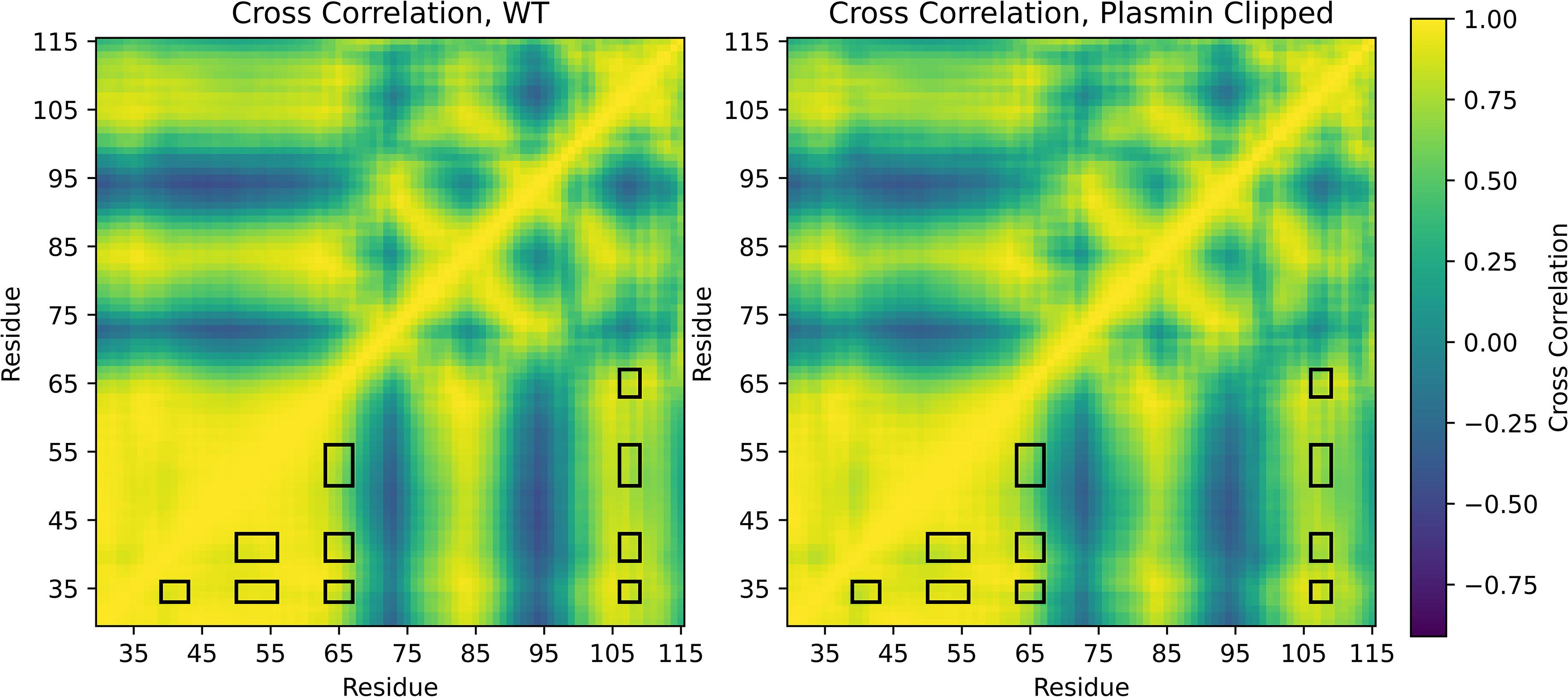

Supplement: Supplementary Fig. 8 — Dynamic cross-correlation plot for DI-DII, with regions of interest highlighted. High cross-correlation between all the regions of interest could indicate the overall stabilising effect of the 106 109 loop displacement on the main R39-R43 epitope. [file mmc8.jpg]
